# A dose-dependent response to MEK inhibition determines hypoblast fate in bovine embryos

**DOI:** 10.1186/s12861-019-0193-9

**Published:** 2019-07-04

**Authors:** Jesica R. Canizo, Amada E. Ynsaurralde Rivolta, Camila Vazquez Echegaray, Mariana Suvá, Virgilia Alberio, Juan F. Aller, Alejandra S. Guberman, Daniel F. Salamone, Ricardo H. Alberio, Ramiro Alberio

**Affiliations:** 1 Estación Experimental Agropecuaria Balcarce, Instituto Nacional de Tecnología Agropecuaria (INTA), Balcarce, Argentina; 2 Laboratorio de Regulación Génica en Células Madre, Departamento de Química Biológica, Facultad de Ciencias Exactas y Naturales, Universidad de Buenos Aires, Buenos Aires, Argentina; 3 Laboratorio de Biotecnología Animal, FAUBA/INPA- CONICET, Universidad de Buenos Aires, Buenos Aires, Argentina; 4 Estación Experimental Agropecuaria Mercedes, Instituto Nacional de Tecnología Agropecuaria (INTA), Corrientes, Argentina; 5 Instituto de Química Biológica (IQUIBICEN), CONICET—Universidad de Buenos Aires, Buenos Aires, Argentina; 6 Departamento de Fisiología y Biología Molecular y Celular, Universidad de Buenos Aires, Facultad de Ciencias Exactas y Naturales, Buenos Aires, Argentina; 7 Facultad de Ciencias Agrarias, Universidad Nacional de Mar del Plata, Mar del Plata, Argentina; 8 School of Biosciences, University of Nottingham, Nottingham, LE12 5RD UK

**Keywords:** Hypoblast, Epiblast, SOX17, NANOG, Bovine embryos, Lineage segregation, PD0325901, LIF, t2iGö, WNT inhibitor

## Abstract

**Background:**

The segregation of the hypoblast and the emergence of the pluripotent epiblast mark the final stages of blastocyst formation in mammalian embryos. In bovine embryos the formation of the hypoblast has been partially studied, and evidence shows that MEK signalling plays a limited role in the segregation of this lineage. Here we explored the role of different signalling pathways during lineage segregation in the bovine embryo using immunofluorescence analysis of NANOG and SOX17 as readouts of epiblast and hypoblast, respectively.

**Results:**

We show that SOX17 starts to be expressed in 16–32-cell stage embryos, whereas NANOG is first detected from 8-cell stage. SOX17 is first co-expressed with NANOG, but these markers become mutually exclusive by the late blastocyst stage. By assessing the expression kinetics of NANOG/SOX17 we show that inhibition of MEK signalling can eliminate SOX17 expression in bovine blastocysts, without altering NANOG expression. Modulation of WNT, PKC and LIF did not affect NANOG expression in the epiblast when used in combination with the ERK inhibitor.

**Conclusions:**

This study shows that SOX17 can be used as a reliable early marker of hypoblast in the bovine, and based on its expression profile we show that the hypoblast segregates in day 7 blastocysts. Furthermore, SOX17 expression is abolished using 1 μM of PD0325901, without affecting the NANOG population in the epiblast. Modulation of WNT, PKC and LIF are not sufficient to support enhanced NANOG expression in the epiblast when combined with ERK inhibitor, indicating that additional signalling pathways should be examined to determine their potential roles in epiblast expansion.

**Electronic supplementary material:**

The online version of this article (10.1186/s12861-019-0193-9) contains supplementary material, which is available to authorized users.

## Background

The bovine embryo transits from the morula to the blastocyst stage between days 5–8 after fertilization. This period is characterized by the emergence of the pluripotent epiblast (EPI) and the segregation of two extraembryonic lineages, the trophectoderm (TE) and the hypoblast (Hypo). The molecular mechanisms of emergence of bovine pluripotent cells and early lineage segregation are not well understood, in part because we have limited knowledge of the expression of key lineage markers in this species. The pluripotency genes OCT4 (*POU5F1*) and SOX2 are expressed from the eight-cell stage, whereas NANOG is first detected in the inner cell mass (ICM) of bovine embryos [1, 2]. GATA6 is one of the earliest hypoblast markers in mice, where it displays a mutually exclusive expression profile with NANOG in the ICM. In bovine, as in human embryos [3, 4], GATA6 and NANOG are co-expressed in many ICM cells (~ 40%), marking hypoblast precursor cells [5]. SOX17 is also expressed in ICM cells, however the expression profile of these cells indicates that this marker identifies cells committed to hypoblast in this species [3, 4, 6, 7]. In bovine embryos SOX17 protein expression has never been reported, however *SOX17* mRNA was detected in presumptive hypoblast cells isolated from blastocysts that also expressed other well-known hypoblast markers *GATA6, FGFR1, HNF4* and *PDGFRα* [8]. Studies exploring the roles of signalling pathways during lineage segregation showed that ERK modulation affects hypoblast development in human, pig and cattle embryos, although the effects are less pronounced than in mouse embryos [3, 5, 9–11]. Moreover ERK inhibition in combination with WNT signalling stimulation was reported to prevent hypoblast segregation in mouse embryos and facilitates embryonic stem cell derivation [12]. However, this approach does not eliminate all hypoblast cells in bovine blastocysts, similar to observations in human and pig embryos, suggesting that other signals may regulate hypoblast segregation [5, 9–11, 13]. In view of these differences among mammals, a better understanding of early gene expression is needed. A deeper understanding of the regulation of signalling pathways in bovine will contribute to the development of better strategies for the isolation of pluripotent cells from bovine blastocysts.

In this study, we investigated the pattern of SOX17 expression during embryo development and used this marker to determine the effects of modulating signalling pathways in the segregation of the hypoblast in bovine blastocysts.

## Results

### Epiblast and hypoblast markers in bovine embryos

**Fig. 1:**
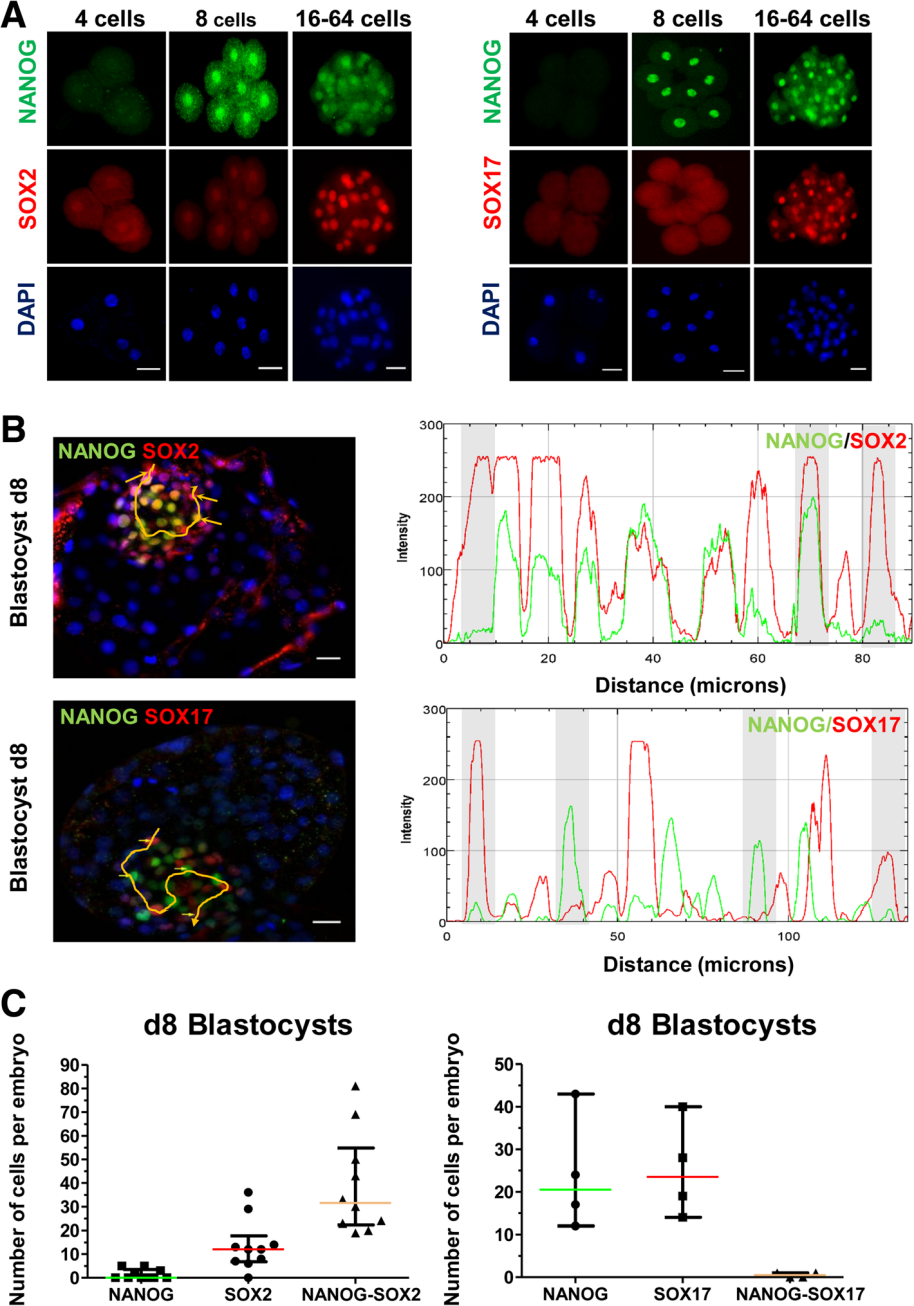
Expression of NANOG, SOX2 and SOX17 in day 2–8 bovine embryos. **a** Immunofluorescence detection of NANOG, SOX2 and SOX17 in different stage embryos. **b** Line scan plot of relative intensity of NANOG, SOX2 and SOX17 in ICM cells of day 8 blastocysts. A line was digitally drawn with FIJI software to measure the fluorescence intensity of different cells and those with and without co-expression of the markers. Cells pointed by an arrow were identified in the scan plot (with grey background) to represent arbitrary fluorescence values. Scale bar: 10 μm. **c** Scatter plot representing number of cells expressing only NANOG, only SOX2, only SOX17 or their co- expressed number of cells in day 8 blastocysts. Data are presented as Median ± IQR

The expression of NANOG, SOX2 and SOX17 was evaluated between days 2–8 on in vitro produced embryos (Fig. 1a and b). SOX2 expression was detected first in 4-cell embryos, with both nuclear and cytoplasmic localization (Fig. 1a). NANOG and SOX2 co-expression was first observed in 8-cell embryos and was maintained until blastocysts at day 8 (Fig. 1a and b). Strong NANOG and SOX2 co-expression was observed in most ICM cells (Fig. 1b and c), however, weak expression was also observed in TE cells of some embryos (see Additional file 1: Figure S1A and S1B). In day 8 blastocysts SOX2 and GATA6 co-localized in most nuclei (see Additional file 1: Figure S2A and S2B). Analysis of immunofluorescence (IF) staining showed that 75 ± 5% of SOX2 positive cells were also NANOG positive (*N* = 10) and 91 ± 5% GATA6 positive (*N* = 3) (Fig. 1c and Additional file 1: Figure S2B). In day 8 hatched blastocysts GATA6 expression was restricted to ICM cells (Additional file 1: Figure S2A), although in some day 8 embryos weak GATA6 expression was also detected in the TE. The high co-expression of SOX2 with NANOG and GATA6 in the ICM indicates that these markers cannot be used to discriminate between epiblast and hypoblast in bovine blastocysts. As SOX17 has been previously used in mouse and human embryos to identify the emergence of the hypoblast [4, 14], we performed a time-course IF analysis from the 4-cell to the blastocyst stage. SOX17 is initially detected in the nucleus of 16–32 cell embryos (Fig. 1a), where it largely overlapped with NANOG. In day 8 blastocysts it became mutually exclusive within the ICM (Fig. 1a and c). In contrast, GATA4 was detected in a very small number of cells in d8 blastocysts (Additional file 1: Figures S2C and S2D) and increased only in d10 embryos (Additional file 1: Figure S2E). These results indicate that SOX17 is a reliable marker for identification of the emergent hypoblast lineage in the bovine embryo.

### In vitro development of bovine embryos in serum free conditions

**Fig. 2:**
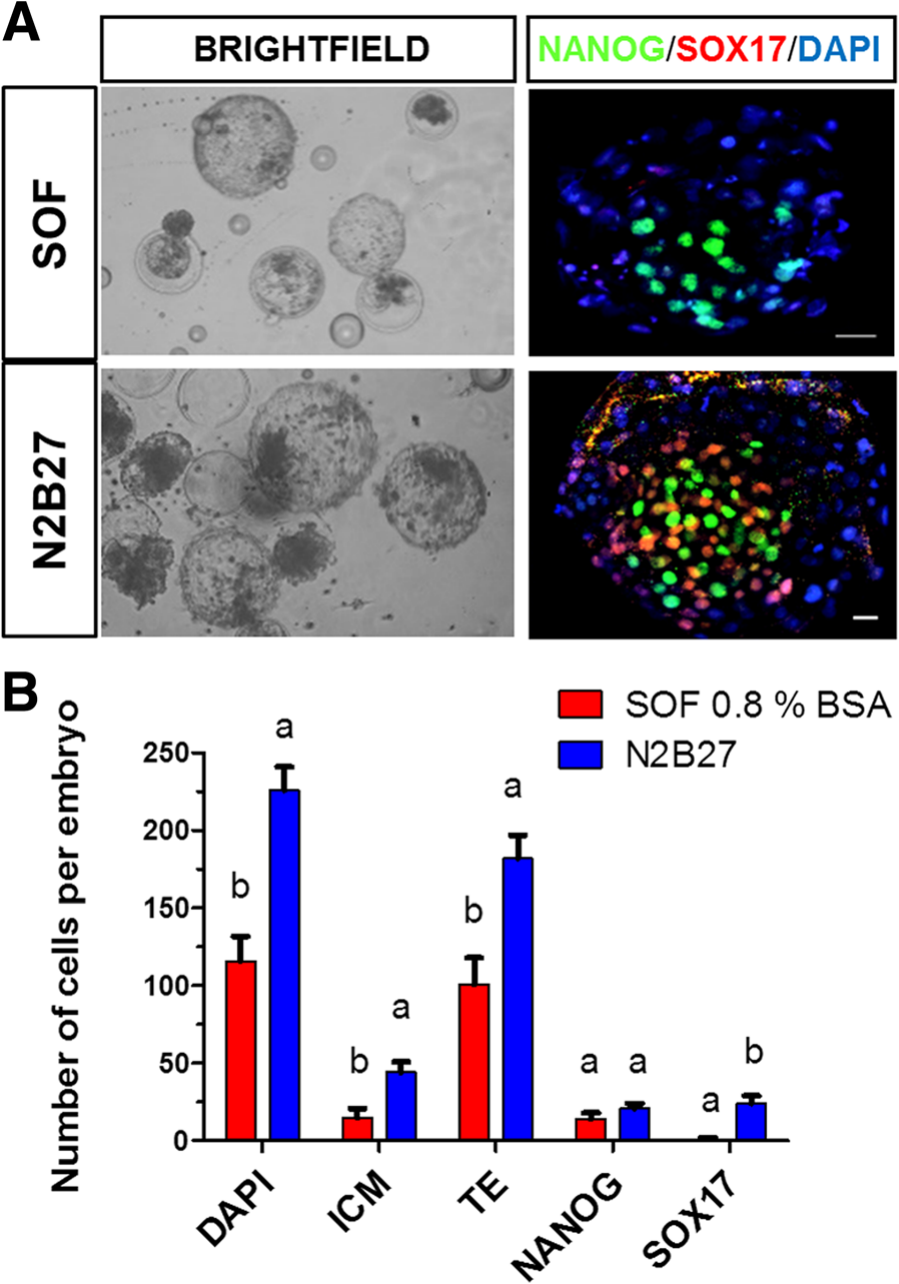
Effect of serum free media in the development of day 7 blastocysts. **a** Representative bright field (left) and immunostaining (right) images of bovine IVF embryos cultured in SOF 0.8% BSA and N2B27. **b** Number of cells per embryo cultured under two conditions. Data are presented as mean ± S.E. The number of embryos analysed was 5 and 7 produced in SOF and N2B27, respectively, from three independent replicates. Different superscripts show significant differences between groups determined by Fisher’s exact test (*p* value< 0.05). Scale bar: 10 μm

To study how specific signalling pathways affect lineage segregation, we tested a serum free culture medium that supports in vitro development to days 8 to 9, as previously shown for pig embryos [9]. Bovine embryos were treated from day 5 to 8, since culturing in N2B27 from the zygote stage had a negative impact on day 2 cleavage rates (data not shown). Culture in SOF and N2B27 media resulted in similar blastocysts rates at days 7 and 8 (Additional file 2: Table S1 and S2), however, embryo quality based on visual criteria was appreciably better in N2B27 than in SOF medium (Fig. 2a). Immunofluorescent staining showed greater number of NANOG and SOX17 positive cells in N2B27 cultured embryos compared to SOF, where SOX17 was almost absent (Fig. 2b). Based on these results we cultured the embryos in N2B27 in subsequent experiments.

### MEK inhibition blocks hypoblast specification

**Fig. 3:**
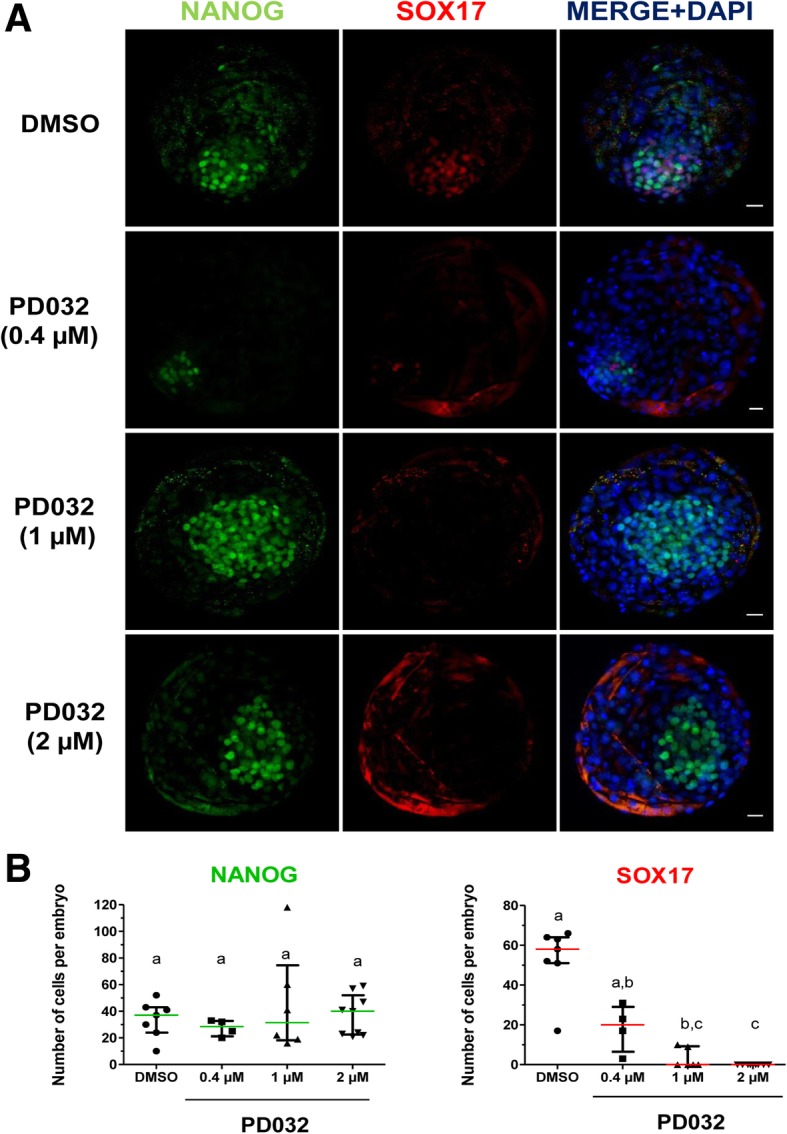
Effect of low dose of MEK inhibitor on NANOG and SOX17 expression in day 8 IVF bovine embryos. **a** Immunostaining detection of NANOG and SOX17 in representative day 8 bovine IVF blastocysts. Each image is a single optical section. **b** Scatter plots showing NANOG and SOX17 positive cells from control and treatments. Data were analysed by Kruskal Wallis and are presented as Median ± IQR. Different superscripts indicate significant difference between groups (*p* < 0.05). N: number of embryos counted after immunofluorescence detection, *N* = 7; 4; 6 and 9 for DMSO, 0.4 μM PD032, 1 and 2 μM PD032 group, respectively. Scale bar: 10 μm

**Fig. 4:**
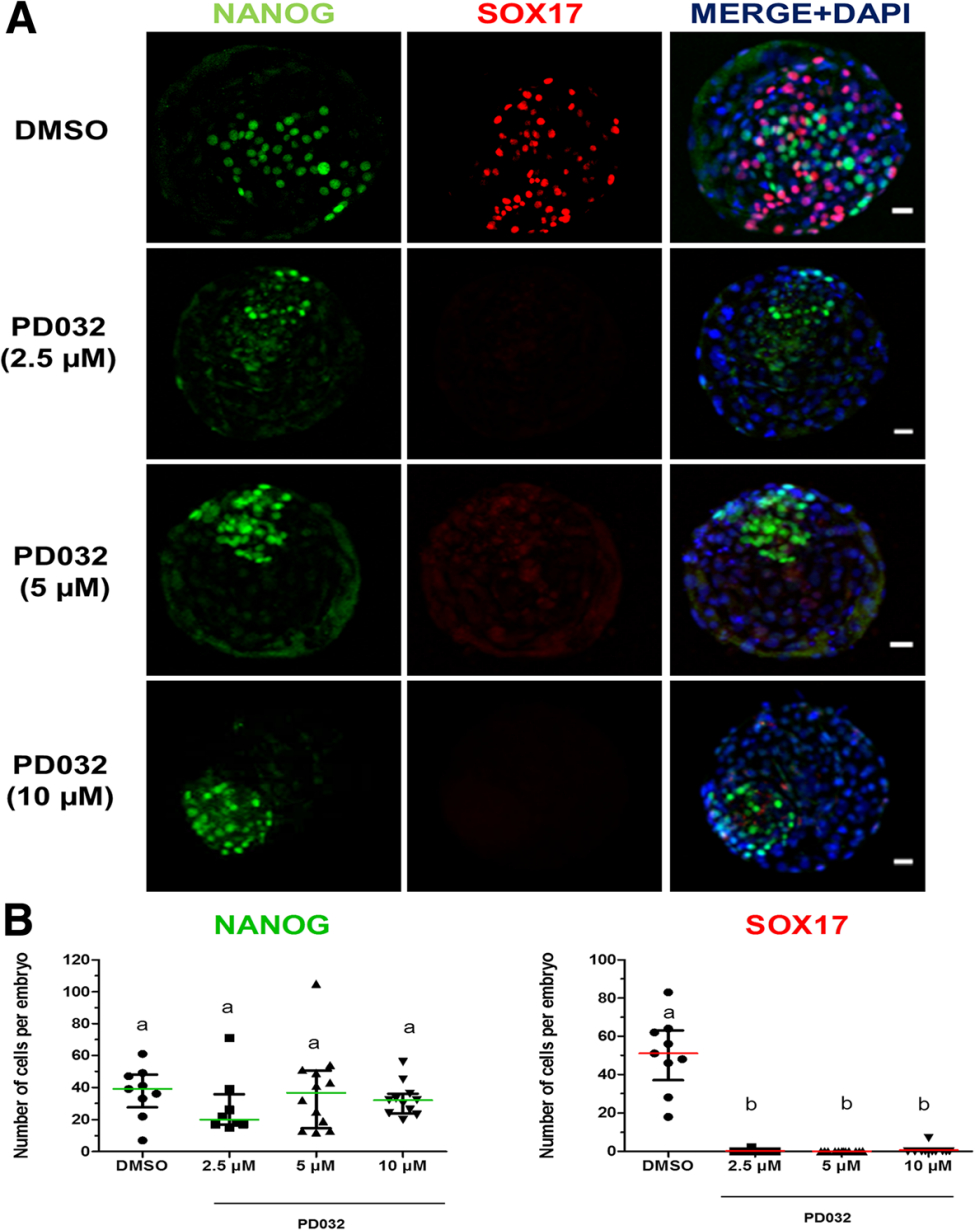
Effect of high dose of MEK inhibitor on NANOG and SOX17 expression in day 8 IVF bovine embryos. **a** Immunostaining detection of NANOG and SOX17 in representative day 8 bovine IVF blastocysts. Each image is a single optical section. **b** Scatter plots showing NANOG and SOX17 positive cells from control and treatments. Data were analysed by Kruskal Wallis and data are presented as Median ± IQR. Different superscripts indicate significant difference between groups (*p* < 0.05). N: number of embryos counted after immunofluorescence detection, *N* = 9; 8; 12 and 11 for DMSO, 2.5 μM PD032, 5 and 10 μM PD032 groups, respectively. Scale bar: 10 μm

To determine the role of MEK signalling during bovine hypoblast specification a dose response to the MEK inhibitor PD0325901 (PD032) was performed (Figs. 3 and 4). At low concentrations of PD032 (0.4–2 μM), development to blastocyst was not affected (Additional file 2: Table S3), however the total number of cells increased in all groups (Table 1). To discriminate between ICM and TE we stained embryos with SOX17 and NANOG (Fig. 3a), to label ICM cells, and counterstained these with DAPI, which was used to determine the number of TE cells. The number of ICM cells was significantly reduced in treated embryos compared to controls; however the number of TE cells significantly increased in all treated groups (Table 1). Analysis of the ICM cells showed that increasing concentrations of MEK inhibition (between 1 and 2 μM) resulted in elimination of SOX17 cells, however the number of NANOG cells remained unchanged (Fig. 3). A further increase in dosage (2.5–10 μM) had no effect in blastocyst development, except for the 10 μM group which showed a higher developmental rate (Additional file 2: Table S4). However the number of TE and ICM cells was reduced in this group compared to lower concentrations (Table 2). The ratio of ICM:TE was reduced in all PD0325901 treatments compared to controls. These results show that a low dose (0.4 μM) of MEK inhibition has a trophic effect on TE, whereas hypoblast segregation is inhibited at higher concentrations (> 1 μM) (Figs. 3 and 4). The number of NANOG positive cells within the ICM did not significantly increase when MEK inhibitor was added at 1 μM compared to 0.4 μM, suggesting that MEK inhibition does not promote epiblast fate, but rather prevents hypoblast segregation. Further increase of MEK inhibitor to 10 μM had a negative effect on TE and ICM compartments (Table 2), although hatching rates reached > 80% in 10 μM treatment with PD0325901.

**Table 1:** Effect of low dose of PD0325901 in bovine embryos

Treatments	N	Total cells	ICM	TE	ICM:TE
DMSO	7	246 ± 24^b^	78 ± 13^a^	166 ± 14^b^	0,47 ± 0,12 ^a^
PD032 (0.4 μM)	4	282 ± 29^a^	58 ± 10^b^	224 ± 20^a^	0,26 ± 0,07 ^b^
PD032 (1 μM)	6	277 ± 27^a^	49 ± 9^b^	228 ± 20^a^	0,21 ± 0,06 ^c^
PD032 (2 μM)	9	270 ± 26^a^	39 ± 7^c^	230 ± 20^a^	0,17 ± 0,05 ^d^

Number of inner cell mass (ICM), trophectoderm (TE) and total cells in embryos cultured under different conditions. Results are from three biological replicates. “N” is the number of embryos analyzed. Values are mean ± S.E. estimated by a GLMM analysis with Poisson distribution and log as link function (for Total Cells, ICM and TE), and with Binomial distribution with logit as link function for (ICM:TE ratios). Different letters denote significant differences, *p* < 0.05

**Table 2:** Effect of high dose of PD0325901 in bovine embryos

Treatments	n	N	Total Cells	ICM	TE	ICM:TE
DMSO	5	9	235 ± 17^a^	86 ± 7^a^	148 ± 14^c^	0,60 ± 0,11 ^a^
PD032 (2.5 μM)	3	8	238 ± 18^a^	28 ± 3^c^	210 ± 20^a^	0,13 ± 0,03 ^b^
PD032 (5 μM)	3	12	252 ± 18^a^	37 ± 3^b^	214 ± 20^a^	0,17 ± 0,03 ^b^
PD032 (10 μM)	3	11	202 ± 14^b^	36 ± 3^b^	82 ± 15^b^	0,16 ± 0,04 ^b^

Number of inner cell mass (ICM), trophoblast (TE) and total cells in embryos cultured under different conditions. “n” is the number of independently IVF runs and “N” is the number of embryos analyzed. Values are mean ± S.E. estimated by a GLMM analysis with Poisson distribution and log as link function (for Total Cells, ICM and TE), and with Binomial distribution with logit as link function for (ICM:TE ratios). Different letters denote significant differences, *p* < 0.05

### t2iGöLIF partially affects SOX17 expression in the bovine ICM

**Fig. 5:**
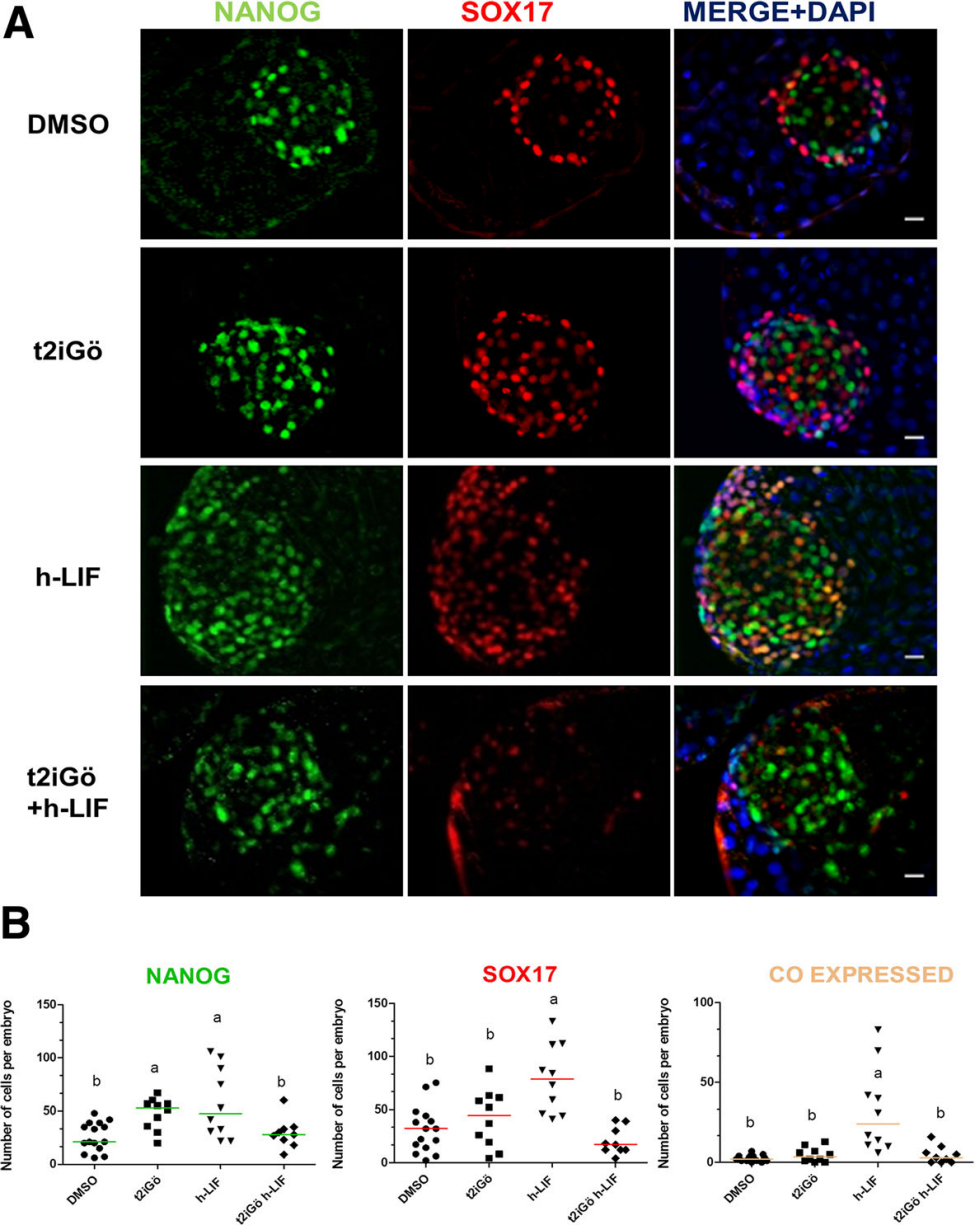
Effect of t2iGö h-LIF on NANOG and SOX17 expression on day 8 bovine embryos. **a** Immunostaining detection of NANOG and SOX17 in representative day 8 bovine IVF blastocysts. Each image is a single optical section. Scale bar: 10 μm. **b** Scatter plots showing number of cells of NANOG, SOX17 and co-expressing cells per embryo. Data were analysed by Kruskall Wallis test and are presented as Median ± IQR. Different superscripts indicate significant difference between groups (*p* < 0.05). N: number of embryos counted after immunofluorescence detection, *N* = 15; 10; 10 and 9 for DMSO, t2iGö, h-LIF, t2iGöLIF groups, respectively

We next studied the crosstalk between MEK signalling and other known pathways involved in pluripotency maintenance in vitro (Fig. 5). We cultured day 5 morulae in N2B27 medium supplemented with PD0325901, CHIR99021, Gö6983, h-LIF and combinations (t2iGöLIF) thereof for 48 and 72 h.

In all conditions analysed, most blastocysts hatched from the zona pellucida, and although the number of blastocyst was not significantly different between DMSO (control), h-LIF and t2iGö + h-LIF, a significantly lower blastocyst rate was obtained in t2iGö-only group (see Additional file 2: Table S5).

All treatments resulted in noticeable increased ICM:TE ratio, however, the total cell number was significantly reduced in t2iGöLIF (Table 3). This was due to a reduction in TE cells, suggesting that t2iGöLIF affects TE proliferation. Finally, the number of ICM cells in t2iGöLIF group was not different to control, however, it increased in t2iGö and LIF (Table 3).

**Table 3:** Effect of t2iGöLIF in bovine embryos

Treatments	n	N	Total cells	ICM	TE	ICM:TE	NANOG:ICM
DMSO	4	15	261 ± 15^a^	53 ± 7^c^	210 ± 20^a^	0,25 ± 0,05	0,45 ± 0,02^b^
t2iGö	3	10	282 ± 17^a^	75 ± 10^b^	198 ± 19^a^	0,39 ± 0,08	0,53 ± 0,04^a^
h-LIF	3	10	281 ± 17^a^	91 ± 12^a^	180 ± 18^b^	0,50 ± 0,08	0,42 ± 0,01^b^
t2iGö h-LIF	3	9	209 ± 13^b^	55 ± 8^c^	142 ± 14^c^	0,38 ± 0,08	0,59 ± 0,02^a^

Number of inner cell mass (ICM), trophectoderm (TE) and total cells in embryos cultured under different conditions. “n” is the number of independently IVF runs and “N” is the number of embryos analyzed. Values are mean ± S.E. estimated by a GLMM analysis with Poisson distribution and log as link function (for Total Cells, ICM and TE), and with Binomial distribution with logit as link function for (ICM:TE and NANOG:ICM ratios). Different letters denote significant differences, *p* < 0.05

To correlate the changes in the number of ICM cells and hypoblast segregation we analysed NANOG and SOX17 expression by IF (Fig. 5a). Embryos treated with t2iGö, and h-LIF had significantly more NANOG cells than controls (Fig. 5b). However, the proportion of NANOG cells in the ICM was significantly greater in t2iGöLIF treatment compared to other groups (Table 3). Of all treatments, only supplementation with h-LIF resulted in increased number of SOX17 cells. In summary, t2iGöLIF had a partial effect in reducing the number of SOX17 positive cells in the bovine ICM, whereas h-LIF had a trophic effect on the hypoblast.

### MEK and WNT signalling inhibition reduce SOX17 expression but do not expand NANOG in the ICM

**Fig. 6:**
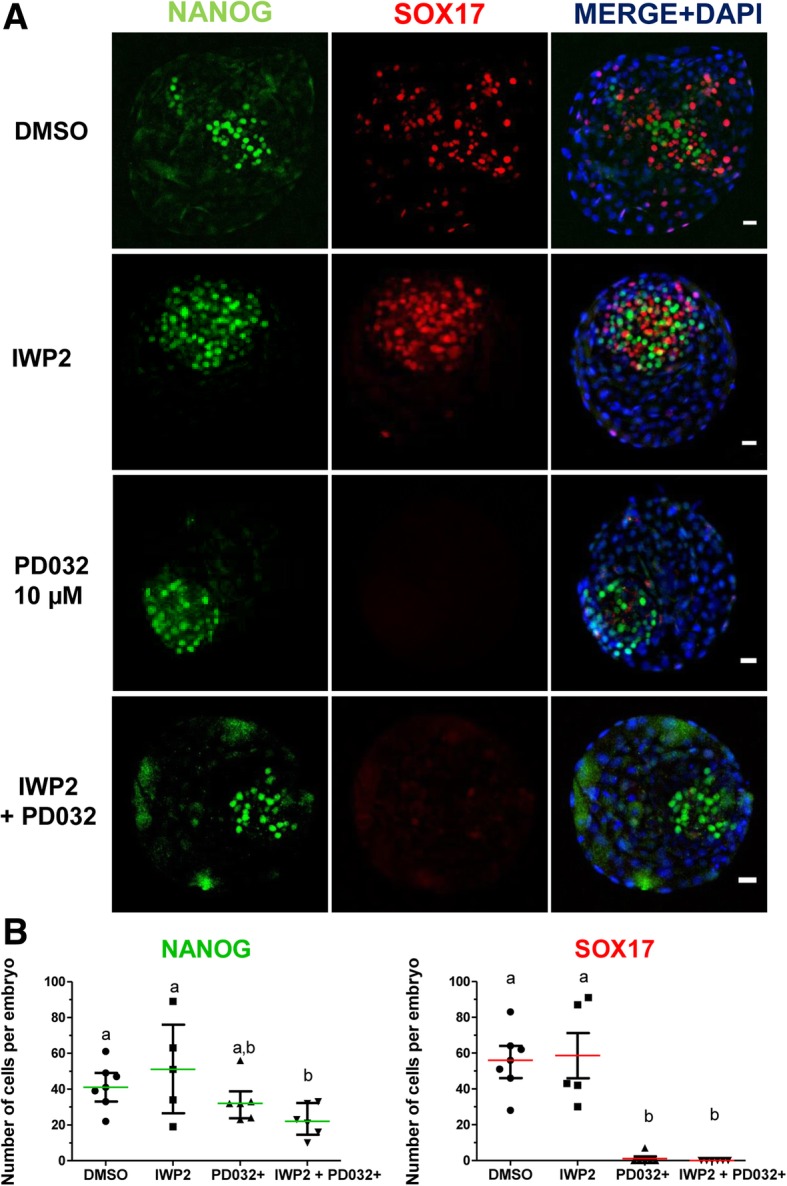
Effect of MEK and Wnt signalling pathways modulation on NANOG and SOX17 expression on day 8 bovine embryos. **a** Immunostaining detection of NANOG and SOX17 in representative day 8 bovine IVF blastocysts. Each image is a single optical section. Scale bar: 10 μm. **b** Scatter plots showing NANOG and SOX17 positive cells in control and treatments. Data were analysed by Kruskal Wallis test and are presented as Median ± IQR. Different superscripts indicate significant difference groups (*p* < 0.05). N: number of embryos counted after immunofluorescence detection, *N* = 7; 5; 6 and 6 for DMSO, 2.5 μM IWP2, 10 μM PD032 and 2.5 μM IWP2 and 10 μM PD032 groups, respectively

We next assessed the effect of MEK (PD032) and WNT inhibition (IWP2) on SOX17 expression, since our previous results showed that inhibition of ERK alone resulted in the absence of SOX17 cells without increase in the number of NANOG positive cells in the ICM (Fig. 6). None of the treatments interfered with the formation of morphologically normal blastocysts (Fig. 6a and see Additional file 2: Table S6), however, 10 μM PD032 resulted in the highest percentage of blastocysts and IWP2 treatment in the lowest (see Additional file 2: Table S6). IWP2 treatment reduced by almost half the percentage of blastocyst at day 8 compared to control (N2B27 with DMSO). IWP2 treatment increased total cell number in blastocysts, mainly due to an increase in TE cells (Additional file 2: Table S6). In contrast, treatment with PD032 (10 μM) decreased the total cell number, which was due to a decrease in the number of ICM cells (Table 4). Although WNT inhibition alone did not affect the number of NANOG and SOX17 positive cells (Fig. 6b), embryos treated with PD032 had almost no SOX17 expressing cells (Fig. 6b). The combination of inhibitors also decreased by half the number of NANOG positive cells, compared to control and WNT inhibition only. Finally, the combined use of ERK and WNT inhibitors did not result in an increase in the number of NANOG positive cells.

**Table 4:** Effect of MEK and Wnt inhibition in bovine embryos

Treatments	n	N	Total cells	ICM	TE	ICM:TE
DMSO	4	7	256 ± 18^b^	97 ± 4^b^	158 ± 19^b^	0,61 ± 0,1 ^b^
IWP2 (2.5 μM)	4	5	302 ± 23ª	118 ± 5^b^	177 ± 22ª	0,70 ± 0,1 ^a^
PD032 (10 μM)	3	6	211 ± 16^c^	34 ± 2^c^	172 ± 21ª	0,20 ± 0,04 ^c^
IWP2 + PD032 (10 μM)	3	6	219 ± 17^c^	25 ± 2^d^	191 ± 24ª	0,13 ± 0,03 ^c^

Number of inner cell mass (ICM), trophectoderm (TE) and total cells in embryos cultured under different conditions. “n” is the number of independently IVF runs and “N” is the number of embryos analyzed. Values are mean ± S.E. estimated by a GLMM analysis with Poisson distribution and log as link function (for Total Cells, ICM and TE), and with Binomial distribution with logit as link function for (ICM:TE ratios). Different letters denote significant differences, *p* < 0.05

## Discussion

### SOX17 marks the emerging hypoblast in the bovine embryo

To gain insight into the mechanism of lineage specification in bovine preimplantation development we assessed the expression of the key developmental regulators NANOG, SOX2 and SOX17, which have been shown to be functionally required for mouse early embryo development. Nanog and Gata6 are co-expressed at the 8-cell stage in the mouse [15], and their expression does not become mutually exclusive until the 32- to 64-cell stage [15]. In bovine embryos, NANOG protein was reported at day 7 (E7) in the ICM although mRNA was detected from the 8-cell stage [1, 2, 5]. RNAseq analysis of in vitro [16–18] and in vivo [19] derived bovine embryos also confirms the presence of NANOG transcripts at the 8-cell stage. We also detected NANOG protein in bovine early embryos (from 8 to 64 cells), and co-expression with SOX2 from 8-cell stage until d8 blastocyst, when NANOG is downregulated earlier than SOX2 in putative hypoblast cells. Our findings are in contrast to previous reports in bovine showing NANOG and SOX2 expression from the morula stage onwards [1, 2]. However, a difference with these studies is that all our IFs were performed in ZP-free embryos. Preliminary IF tests with ZP-enclosed embryos failed to provide consistent staining for any of the markers tested, probably due to reduced permeability to the antibodies (Additional file 1: Figure S3). NANOG staining showed very weak signal, whereas strong background accompanied SOX2 and SOX17 staining. It is therefore possible that the presence of ZP may interfere with the detection of low abundance proteins, like NANOG, in early embryonic stages of bovine embryos.

As SOX2 and GATA6 were co-expressed in most bovine ICM cells at day 8, we studied SOX17 and GATA4 expression, as alternative markers of hypoblast. In mice, Sox17 expression starts at the 32-cell stage and marks the nascent hypoblast. These cells then express Gata4 at the 64-cell stage [14]. In human, SOX17 is initially detected in 32-cell stage embryos and also precedes GATA4 expression [4]. Here we show that in bovine embryos SOX17 is localized in the nucleus from 16 to 32 cell stage and becomes mutually exclusive with NANOG in putative hypoblast cells on day 7/8 blastocysts. As in human and mouse embryos, GATA4 is restricted to hypoblast cells of day 8 or older bovine blastocysts, indicating that SOX17 precedes GATA4 activation. Together, our analysis shows that SOX17 marks the nascent hypoblast, and that these cells segregate from a NANOG positive population in the ICM.

### Effect of signalling modulation on bovine embryo development and hypoblast segregation

The use of 2i accelerates bovine blastocyst development, promotes expression of epiblast markers *NANOG* and *SOX2* and reduces expression of the hypoblast marker *GATA4* [10]. Suppression of hypoblast segregation can also be achieved using a MEK inhibitor (PD0325901) at high concentrations (10 μM or more), which could be due to off target effects on unknown signalling pathways [11]. Our dose response experiment shows that low dose of MEK inhibition (0.4–2 μM) increases the number of TE cells, whilst expression of SOX17 was almost eliminated at 1 μM and higher, indicating that in bovine embryos a modest increase (~ 2.5-fold) of PD0325901 from the concentration used in mouse, is sufficient to abrogate hypoblast segregation. The mechanism underlying the elimination of SOX17 expression in bovine embryos is still unclear, and more studies are needed to clarify the effect of MEK inhibition in the hypoblast and the potential effects of high concentration of MEK inhibitor on other intracellular signalling pathways. Notably, our study shows for the first time a trophic effect of MEK inhibition in the bovine TE, in contrasts to findings in mouse embryos [20], highlighting potential species differences in the role of ERK signalling during TE development.

To gain further understanding on what other signals may interfere with hypoblast segregation in bovine embryos we used more complex culture conditions previously reported for derivation of naïve h-ESC, consisting of N2B27 medium supplemented with titrated 2i + LIF and the PKC inhibitor Gö6983. We found that t2iGö alone yielded lower blastocyst rates, despite the increased size of the ICM in these embryos. The ICM maintained similar proportion of hypoblast cells, however when LIF was added, SOX17 expression was significantly reduced. These observations are consistent with findings in mouse embryos showing that PKC inhibition delays the basal rate of cavitation [21] and participates in trophectoderm fate [22]. When LIF is added to this cocktail, blastocyst yields increase, probably as a result proliferative and anti-apoptotic effects of JAK/STAT3 and PI3K/Akt, respectively [23, 24]. LIF alone, however, promotes expansion of hypoblast in bovine embryos, as observed in mouse embryos [25]. We also detected higher number of NANOG positive cells under these conditions, suggesting that LIF has a trophic effect in the bovine ICM by increasing NANOG and SOX17 positive cells at day 8. These observations are consistent with previous findings in bovine embryos showing that inhibition of JAK/STAT interferes with ICM formation, but trophectoderm commitment was not affected [26]. Additionally, in line with our results, JAK inhibition in bovine embryos leads to reduction in both epiblast (*NANOG* and *FGF4*) and hypoblast (*SOX1*7 and *PDGFRα*) transcripts, and other naïve pluripotency-related genes (*KLF4*, *TFCP2L1*) and STAT3 [26]. Together, these observations suggest that the combined use of an inhibitor of the TE pathway (PKC) and an agonist of TE inhibition (WNT) results in reduced blastocyst development and increased ICM:TE ratio, in part due to the reduced specification of TE lineage. When LIF is added to this cocktail (t2iGö + LIF) it promotes expansion of NANOG cells in the ICM, whilst SOX17 expression is reduced, possibly due to the inhibition of MAPK signalling.

Finally, we analysed ERK and WNT inhibition during hypoblast segregation. In marmoset, hypoblast differentiation is reduced by ERK and WNT inhibition, whilst NANOG expression in the epiblast increases [27]. WNT inhibition combined with FGF supplementation is also used in primates, including human, for the establishment of both embryonic and induced pluripotent stem cells (PSCs) [28]. The WNT signalling pathway is active in bovine blastocysts, and canonical WNT stimulation inhibits TE formation, whereas WNT inhibition by DKK1 promotes TE and hypoblast proliferation [29, 30]. Consistent with these results we show that WNT inhibition with IWP2 also promotes TE proliferation, however the number of SOX17 positive cells was unaffected and the number of NANOG positive cells increased. The differences with the previous studies could be due to the different efficacy of WNT inhibition by DKK1 and IWP2 over the course of the treatment.

## Conclusion

**Fig. 7:**
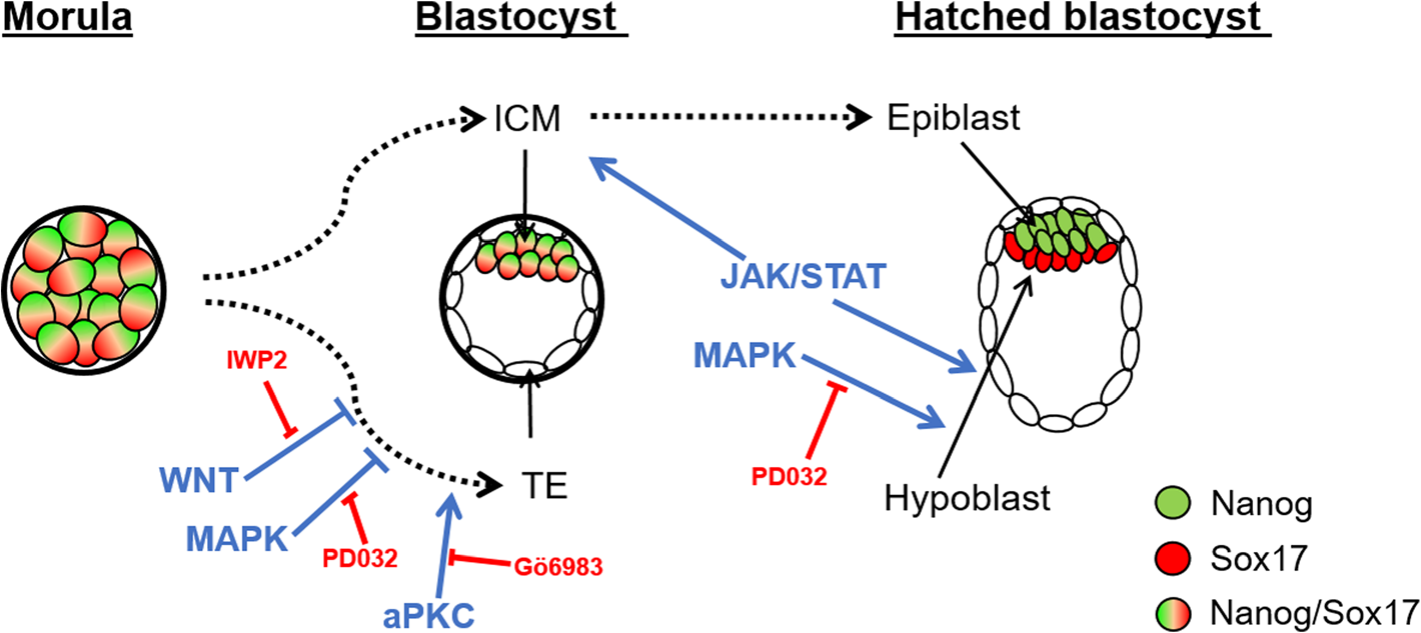
Summary of the signalling pathways operating during bovine embryo development. Diagrammatic representation of the signalling pathways investigated in our study. Small molecule inhibitors are indicated with red letters. Stimulatory pathways are indicated with arrows, and inhibitory pathways with blunt ends

This study shows that SOX17 can be used as a reliable early marker of hypoblast in the bovine, and based on its expression profile we show that the hypoblast segregates in day 7 blastocysts. Furthermore, SOX17 expression is abolished using 1 μM of PD0325901, without affecting the NANOG population in the epiblast. Modulation of WNT, PKC and LIF are not sufficient to support enhanced NANOG expression in the epiblast when combined with ERK inhibitor (Fig. 7), indicating that additional signalling pathways should be examined to determine their potential roles in epiblast expansion.

## Methods

Research protocols followed the guidelines stated in the Guide for the Care and Use of Agricultural Animals in Agricultural Research and Teaching.

### Chemicals and reagents

All chemicals and reagents were purchased from Sigma-Aldrich unless otherwise stated.

### Oocyte collection

Ovaries from cycling beef heifers (*Bos taurus*) were collected from local abattoirs and transported to the laboratory at 25–30 °C. Cumulus oocyte complexes (COCs) were aspirated from follicles of 2 to 8 mm diameter using a 21-gauge needle and collected into Hepes-buffered Tyrode’s albumin (Hepes-TALP) [31]. COCs with homogeneous ooplasm and more than three complete layers of cumulus cells, corresponding to grades 1 and 2 according to de Loos et al., [32], were selected under a stereomicroscope and washed 3 times in Hepes-TALP.

### In vitro maturation

The selected COCs were in vitro matured in 100 μl droplets of bicarbonate-buffered TCM-199 (31100–035; Gibco), containing 10% fetal bovine serum (013/07; Internegocios S.A.), 10 mg/ml follicle-stimulating hormone (NIH-FSH-P1, Folltropin, Bioniche), 0.3 mM sodium pyruvate (P2256), 100 mM cysteamine (M9768), and 2% antibiotic-antimycotic (ATB, 15240–096; Gibco); and covered in mineral oil (M8410). The oocytes were incubated for 22 h at 39 °C under 6.5% CO_2_ in humidified air.

### IVF procedures and embryo culture

Embryos used in Figs. 1, 2, 3, 4 and 6 were produced following the protocol described by Hiriart et al.*,* [33]. For embryos used in Fig. 5 embryos were produced according to the protocol detailed in [34]. Experiments between the two laboratories were not compared to each other. All treatments were carried out in N2B27 base medium from day 5 onwards.

In vitro fertilization was performed following the protocol by Hiriart et al.*,* (2013). Frozen semen was thawed and washed twice by centrifugation in Brackett-Oliphant medium (BO) supplemented with 5 mM caffeine (C4144) and 20 IU/mL heparin (H3149). Sperm dilution was adjusted to 16 × 10^6^ spermatozoa/ml adding BO containing 10 mg/mL bovine serum albumin (A6003). Sperm was then co-incubated with IVM COCs in 100 μl droplets of the sperm suspension for 5 h at 39 °C under 6.5% CO_2_ in humidified air. Presumptive zygotes were washed three times in Hepes-TALP and cumulus cells were removed by vortexing for 2 min in hyaluronidase (H4272) (1 mg/mL in Dulbecco’s PBS). Denuded presumptive zygotes were cultured in 50 μl droplets of Synthetic Oviductal Fluid (SOF) supplemented with foetal bovine serum (FBS) at 2.5% and covered with mineral oil, at 39 °C under 6.5% CO_2_ in humidified air. Cleavage was evaluated on day 2 and development to blastocyst on days 7 and 8. Embryos from morula to blastocyst stage were cultured in 20 μl droplets of N2B27 medium and in those experiments that included inhibitors or DMSO, medium was changed after 48 h.

Embryos used to generate Fig. 5 were produced according to the protocol detailed in [34]. Briefly, COCs were repeatedly pipetted until 2–3 layers of granulosa cells were left around the oocyte. Groups of 40–50 COCs were then incubated in 0.5 ml of sperm at a concentration of 0.5–1.0 × 10^6^ sperm/ml of fertilization medium and cultured for 20 h at 39 °C in a humidified incubator with an atmosphere of 5% CO2 in 95% N_2_. After 20 h (day 1), embryos were washed twice in HEPES buffered modified synthetic oviductal fluid (H-SOF) medium and presumptive zygotes were transferred into droplets with BBH7 medium [35] until day 5. Embryo culture was carried out at 39 °C, in a humidified incubator with a gaseous atmosphere of 5% CO2, 5% O2 and 90% N_2_. Treatments were carried out from day 5 in droplets of N2B27 medium. DMSO was added to control group to the level used for the inhibitors.

### Serum free culture of bovine embryos

Between days 5–7/8 embryos were cultured in two serum free media i) Synthetic Oviductal Fluid medium, supplemented with 0.8% of BSA and ii) N2B27 stem cell culture medium. For a final volume of 5 ml, N2B27 was made by adding 2.5 ml of DMEM/F12 (Thermo Fisher Scientific catalogue number 12500–062), 2.5 ml of Neurobasal medium (GIBCO, Thermo Fisher Scientific, catalogue number 21103–049), 25 μl of N2 100X supplement (Thermo Fisher Scientific, catalogue number 17502048), 50 μl of B27 50X supplement (Thermo Fisher Scientific, catalogue number 112587010), 5 μl of insulin (10 mg/ml, Sigma Aldrich catalogue number I9278), 0.8% of BSA and 50 μl of 100X penicillin/streptomycin solution. Medium was prepared, aliquoted and frozen at − 20 °C until use.

We performed IVF runs in triplicate for immunostaining analysis (fixing the hatched embryos at day 8).

### Modulation of signalling pathways

MEK inhibition dose response curve: from day 5, embryos were cultured in N2B27 with DMSO (control) or in N2B27 with 0.4–10 μM of PD0325901 (TOCRIS, catalogue number 4192). Development was evaluated at day 7 and 8 and embryos were classified as BL: blastocyst, EB: expanded blastocyst, BHing: hatching blastocyst and BHed: hatched blastocyst. Hatched embryos were fixed with 4% of PFA at day 8 prior to immunostaining analysis. For MEK and Wnt inhibition experiments day 5 embryos were cultured in N2B27 with i) 10 μM of PD0325901, ii) 2.5 μM of IWP2 (Stemgent, catalogue number 04–0034), and iii) the combination of 10 μM PD0325901 and 2.5 μM IWP2. For t2iGö + LIF, t2iGö and LIF, a modified concentration of 1 μM CHIR (TOCRIS, catalogue number 4423), 1 μM of PD0325901, 2 μM of PKC inhibitor Gö6983 (AXON MEDCHEM,) and 20 ng/ml of h-LIF (Peprotech, catalogue number 300–05) were used.

### IF of whole embryos

Immunostaining of bovine embryos without ZP was performed according to the protocol detailed in Nichols et al.*,* [20]. Primary antibodies used in this study were: rabbit anti-human NANOG (dilution 1:200, catalogue number 500-P236, Peprotech), goat anti-human SOX17 (dilution 1:200, catalogue number AF1924, R&D company), rabbit anti-human GATA6 (dilution 1:100, catalogue number 22600, ABCAM), goat anti-mouse C terminus Gata4 (dilution 1:100, catalogue number sc-1237, Santa Cruz) and goat anti-human SOX2 (dilution 1:200, catalogue number sc-17320, Santa Cruz). Appropriate secondary antibodies were used as reported before [36]. Embryos were mounted on slides with Fluoroshield with DAPI mounted reagent (catalogue number F6057, Sigma Aldrich).

Images from immunostained embryos were taken under an epifluorescence microscope and were analysed using the FIJI software (ImageJ). In all cases, background was subtracted and generally a Gaussian filter was applied. A representative negative control embryo, in which the level of the background was established, is shown in Additional file 1: Figure S4. The function multichannel plot profile from BAR (Broadly Applicable Routines) tools in FIJI was used to detect co- localization of fluorescent signals. For counting cells, we created masks for each channel and used the tool analyse particles.

### Statistical analysis

Statistical analysis was carried out using InfoStat software [37]. Fluorescence intensity expressed in Arbitrary Units (A.U.) was analysed through Student’s t test when two mean values were compared (Additional file 1: Figures S1B and S4B). Number of NANOG and SOX17 cells per embryo (by definition, a response variable following a Poisson distribution) was compared using a non-parametric ANOVA (Kruskal Wallis, KW). A Generalized Linear Mixed Models assay (GLMM) with Poisson family and log link function was used for the total number of cells (DAPI), ICM and TE number of cells per embryo analysis. GLMM were applied when median comparisons were around the significance level but higher, in order to resolve differences not detected previously in KW test due to high dispersion from random effects, this was the case of DAPI, ICM and TE counts per embryo. In GLMM the “type of medium” was analysed as fixed effect and the “number of IVF run” was used as random effect due to random variability between batches of ovaries, days and culture conditions. Appropriate link functions were applied for the analyses; results are presented on the observed scale after application of the inverse link function.

Proportions (percentage of cells, by definition a response variable following a Binomial distribution) were also analysed by GLMM but with Binomial distribution and with logit as link function as indicated in the footnotes of the tables. Differences between treatments were considered significant at *p* value < 0.05 and detected by DGC contrast [38]. InfoStat implements a friendly interface of the R platform for the estimation of the generalized linear mixed models through the functions “glm” from the “*stats” library* and “glmer” from the “lme4” library.

Results from KW test were presented as Figures (Scatter plot with median and IQR, Figs. 3b, 4b, 5b and 6b) and GLMM results, estimated averages +/− S.E., were reported as tables (Tables 1, 2, 3, 4).

When embryo developmental rates were analysed for two groups (two type of medium), a Fisher’s exact test was applied (Additional file 2: Tables S1 and S2). For more than two groups a GLMM were also used for the Poisson response variable “number of embryo per treatment” and for the Binomial response variable “production ratio: number of embryo per number of presumptive zygotes”. Here, “type of embryo culture medium” was used as fixed effects and “IVF number of run” as random effects; log link function was used for Poisson distribution. Logit was used as link function for Binomial distributions and is indicated in each table footnote. Differences between treatments were considered significant at *p* value < 0.05 and detected by DGC contrast [41]. Estimated averages +/− S.E. are reported in Tables (see Additional file 2: Tables S3, S4, S5 and S6).

## Additional files

Additional file 1: **Figure S1.** Expression of NANOG and SOX2 in day 8 bovine embryos. **Figure S2.** Expression of NANOG, SOX2, GATA4, GATA6 in day 8 and 10 bovine embryos. **Figure S3.** Expression of NANOG (N=4), SOX2 (N=2) and SOX17 (N=2) in bovine embryos with ZP. **Figure S4.** A representative negative control of the immunofluorescence protocol. (DOCX 2405 kb)

Additional file 2: **Table S1.** Blastocyst development rates at day 7 in SOF and N2B27. **Table S2.** Blastocyst development rates at day 8. **Table S3.** Blastocyst development rates of bovine embryos cultured in low doses of PD0325901. **Table S4.** Blastocyst development rates of bovine embryos cultured in high doses of PD0325901. **Table S5.** Blastocyst development rates of bovine embryos cultured in t2iGö+LIF. **Table S6.** Effect of Wnt and MEK inhibitors on bovine blastocyst development. (DOCX 38 kb)

## Data Availability

The datasets used and/or analysed during the current study are available from the corresponding author on reasonable request.

## Abbreviations

2i: MEK and GSK3β inhibitors
BAR: A collection of Broadly Applicable Routines
BHed: Hatched blastocyst
BHing: Blastocyst hatching
BL: Blastocyst
Ct: Threshold cycle
DGC: Di Rienzo, Guzmán and Casanoves
EB: Expanded blastocyst
Epi: Epiblast
FBS: Foetal bovine serum
FGF: Fibroblast growth factor
GLMM: Generalized linear mixed models
h-ESC: Human embryonic stem cells
ICM: Inner cell mass
IF: Immunofluorescence
IQR: Interquartilic range
IVF: In vitro fertilization
IWP2: Wnt inhibitor
LIF: Leukaemia inhibitory factor
LSD: Least significant difference
m-ESC: Mouse embryonic stem cells
No: Initial number of molecules
PCR: Polymerase chain reaction
PD032: PD0325901, MEK inhibitor
PFA: Paraformaldehyde
PSC: Pluripotent stem cells
qPCR: Quantitative PCR
TE: Trophectoderm
TFs: Transcription factors

## References

[1] Kuijk EW, Du Puy L, Van Tol HTA, Oei CHY, Haagsman HP, Colenbrander B (2008). Differences in early lineage segregation between mammals. Dev Dyn.

[2] Khan DR, Dubé D, Gall L, Peynot N, Ruffini S, Laffont L (2012). Expression of pluripotency master regulators during two key developmental transitions: EGA and early lineage specification in the bovine embryo. PLoS One.

[3] Roode M, Blair K, Snell P, Elder K, Marchant S, Smith A (2012). Human hypoblast formation is not dependent on FGF signalling. Dev Biol.

[4] Niakan KK, Eggan K (2013). Analysis of human embryos from zygote to blastocyst reveals distinct gene expression patterns relative to the mouse. Dev Biol.

[5] Kuijk EW, Tol v, LT a, Van de Velde H, Wubbolts R, Welling M, Geijsen N (2012). The roles of FGF and MAP kinase signaling in the segregation of the epiblast and hypoblast cell lineages in bovine and human embryos. Development.

[6] Petropoulos S, Edsgärd D, Reinius B, Deng Q, Panula SP, Codeluppi S (2016). Single-cell RNA-Seq reveals lineage and X chromosome dynamics in human preimplantation embryos. Cell.

[7] Stirparo Giuliano G., Boroviak Thorsten, Guo Ge, Nichols Jennifer, Smith Austin, Bertone Paul (2018). Integrated analysis of single-cell embryo data yields a unified transcriptome signature for the human pre-implantation epiblast. Development.

[8] Negrón-Pérez VM, Rodrigues LT, Mingoti GZ, Hansen PJ (2018). Role of ROCK signaling in formation of the trophectoderm of the bovine preimplantation embryo. Mol Reprod Dev.

[9] Rodríguez A, Allegrucci C, Alberio R (2012). Modulation of pluripotency in the porcine embryo and iPS cells. PLoS One.

[10] Harris D, Huang B, Rn Oback B (2013). Inhibition of MAP2K and GSK3 signaling promotes bovine blastocyst development and epiblast-associated expression of pluripotency factors 1. Biol Reprod.

[11] McLean Z, Meng F, Henderson H, Turner P, Oback B (2014). Increased MAP kinase inhibition enhances epiblast-specific gene expression in bovine blastocysts. Biol Reprod.

[12] Nichols J, Smith A (2009). Naive and primed pluripotent states. Cell Stem Cell.

[13] Ramos-Ibeas P, Nichols J, Alberio R (2018). States and origins of mammalian embryonic pluripotency in vivo and in a dish. Current topics in developmental biology.

[14] Niakan KK, Ji H, Maehr R, Vokes SA, Rodolfa KT, Sherwood RI (2010). Sox17 promotes differentiation in mouse embryonic stem cells by directly regulating extraembryonic gene expression and indirectly antagonizing self-renewal. Genes Dev.

[15] Plusa B, Piliszek A, Frankenberg S, Artus J, Hadjantonakis A-K (2008). Distinct sequential cell behaviours direct primitive endoderm formation in the mouse blastocyst. Development.

[16] Graf A, Krebs S, Zakhartchenko V, Schwalb B, Blum H, Wolf E (2014). Fine mapping of genome activation in bovine embryos by RNA sequencing. Proc Natl Acad Sci U S A.

[17] Graf A, Krebs S, Heininen-Brown M, Zakhartchenko V, Blum H, Wolf E (2014). Genome activation in bovine embryos: review of the literature and new insights from RNA sequencing experiments. Anim Reprod Sci.

[18] Lavagi I, Krebs S, Simmet K, Beck A, Zakhartchenko V, Wolf E (2018). Single-cell RNA sequencing reveals developmental heterogeneity of blastomeres during major genome activation in bovine embryos. Sci Rep.

[19] Jiang Z, Sun J, Dong H, Luo O, Zheng X, Obergfell C (2014). Transcriptional profiles of bovine in vivo pre-implantation development. BMC Genomics.

[20] Nichols J, Silva J, Roode M, Smith A (2009). Suppression of Erk signalling promotes ground state pluripotency in the mouse embryo. Development.

[21] Stachecki JJ, Randall Armant D (1996). Transient release of calcium from inositol 1,4,5-trisphosphate-specific stores regulates mouse preimplantation development. Development.

[22] Plusa B, Frankenberg S, Chalmers A, Hadjantonakis A-K, Moore CA, Papalopulu N (2005). Downregulation of Par3 and aPKC function directs cells towards the ICM in the preimplantation mouse embryo. J Cell Sci.

[23] Niwa H, Ogawa K, Shimosato D, Adachi K (2009). A parallel circuit of LIF signalling pathways maintains pluripotency of mouse ES cells. Nature.

[24] Pera MF, Tam PPL (2010). Extrinsic regulation of pluripotent stem cells. Nature.

[25] Morgani SM, Brickman JM (2015). LIF supports primitive endoderm expansion during pre-implantation development. Development.

[26] Meng F, Forrester-Gauntlett B, Turner P, Henderson H, Oback B (2015). Signal inhibition reveals JAK/STAT3 pathway as critical for bovine inner cell mass development. Biol Reprod.

[27] Boroviak T, Loos R, Lombard P, Okahara J, Behr R, Sasaki E (2015). Lineage-specific profiling delineates the emergence and progression of naive pluripotency in mammalian embryogenesis. Dev Cell.

[28] Wu J, Okamura D, Li M, Suzuki K, Luo C, Ma L (2015). An alternative pluripotent state confers interspecies chimaeric competency. Nature.

[29] Denicol AC, Dobbs KB, McLean KM, Carambula SF, Loureiro B, Hansen PJ (2013). Canonical WNT signaling regulates development of bovine embryos to the blastocyst stage. Sci Rep.

[30] Denicol AC, Block J, Kelley DE, Pohler KG, Dobbs KB, Mortensen CJ (2014). The WNT signaling antagonist Dickkopf-1 directs lineage commitment and promotes survival of the preimplantation embryo. FASEB J.

[31] Bavister BD (1977). Yanagimachi. The effects of sperm extracts and energy sources on the motility and acrosome reaction of hamster spermatozoa in vitro. Biol Reprod.

[32] de Loos F, van Vliet C, van Maurik P, Kruip TA (1989). Morphology of immature bovine oocytes. Gamete Res.

[33] Hiriart MI, Bevacqua RJ, Canel NG, Fernández-Martín R, Salamone DF (2013). Production of chimeric embryos by aggregation of bovine egfp eight-cell stage blastomeres with two-cell fused and asynchronic embryos. Theriogenology.

[34] Maalouf WE, Alberio R, Campbell KHS (2008). Differential acetylation of histone H4 lysine during development of in vitro fertilized, cloned and parthenogenetically activated bovine embryos. Epigenetics.

[35] Block J, Bonilla L, Hansen PJ (2010). Efficacy of in vitro embryo transfer in lactating dairy cows using fresh or vitrified embryos produced in a novel embryo culture medium1. J Dairy Sci.

[36] Kobayashi T, Zhang H, Tang WWC, Irie N, Withey S, Klisch D (2017). Principles of early human development and germ cell program from conserved model systems. Nature.

[37] Di Rienzo JA, Casanoves F, Balzarini MG, Gonzalez L, Tablada M, Robledo CW (2017). Infostat software student version.

[38] Di RJA, Guzmán AW, Casanoves F (2002). A multiple-comparisons method based on the distribution of the root node distance of a binary tree. J Agric Biol Environ Stat.

