# Supplementary material for: A dose-dependent response to MEK inhibition determines hypoblast fate in bovine embryos
Source: BMC Dev Biol. 2019 Jul 4;19:13. doi: 10.1186/s12861-019-0193-9 (PMC6610975; doi:10.1186/s12861-019-0193-9)
Supplement: Supplementary file 1 — Figure S1. Expression of NANOG and SOX2 in day 8 bovine embryos. Figure S2. Expression of NANOG, SOX2, GATA4, GATA6 in day 8 and 10 bovine embryos. Figure S3. Expression of NANOG (N=4), SOX2 (N=2) and SOX17 (N=2) in bovine embryos with ZP. Figure S4. A representative negative control of the immunofluorescence protocol. (DOCX 2405 kb) [file 12861_2019_193_MOESM1_ESM.docx]

**Additional File 1, (Canizo *et al.,)***

**Figure S1: Expression of NANOG and SOX2 in day 8 bovine embryos.** Immunofluorescence detection in ICM and TE of bovine blastocysts. The image is a single optical section of NANOG and SOX2 co-staining. (B) Bar plot of NANOG and SOX2 fluorescence intensity (A.U.: arbitrary units) in ICM and TE. Data are presented as mean ± SEM (N=3), different letters denote significant differences detected by a t-test (p value< 0.05). Scale bar: 10 µm

**Figure S2: Expression of NANOG, SOX2, GATA4, GATA6 in day 8 and 10 bovine embryos.** Immunofluorescence detection in bovine pre-implantation embryos. Each image is a single optical section. (A) SOX2 and GATA6 co-staining in d8 embryos. (B) Scatter plot of the number of cells per embryo that expresses only SOX2, only GATA6 and co- expresses both genes (N=3) (left). Line scan plot of relative intensity of SOX2 and GATA6 in ICM cells of day 8 blastocysts (right). (C) NANOG and GATA4 co-staining in d8 embryos (arrows: GATA4+ cells). (D) Line scan plot of relative intensity of NANOG and GATA4 in ICM cells of day 10 blastocyst. (E) Scatter plot of the number of cells staining positive for NANOG and GATA4, or both markers together in d8 (N=8) and d10 (N=3) bovine embryos. Median and I.Q. are also indicated. Scale bar: 10 µm.

**NANOG**

**SOX2**

**SOX17**

**DAPI**

**DAPI**

**NANOG**

**Figure S3: Expression of NANOG (N=4), SOX2 (N=2) and SOX17 (N=2) in bovine embryos with ZP.** Immunofluorescence detection in day 4 (16 cells) embryos. Each image is a single optical section of NANOG co- stained with SOX2 or SOX17, as indicated. Scale Bar: 10 µM

**Figure S4: A representative negative control of the immunofluorescence protocol.** (A) A d8 blastocyst cultured in N2B27 with DMSO and stained with secondary antibody only. Each image is a single optical section of fluorescence at channels where NANOG was detected (green) and SOX17 was detected (red) as indicated. (B) Quantification of background level in arbitrary units (A.U.) of fluorescence in three aleatory sections of embryo and image background, different letters denote significant differences determined by t-test (p value< 0.05). Scale Bar: 10 µM.
