# Supplementary material for: A dose-dependent response to MEK inhibition determines hypoblast fate in bovine embryos
Source: BMC Dev Biol. 2019 Jul 4;19:13. doi: 10.1186/s12861-019-0193-9 (PMC6610975; doi:10.1186/s12861-019-0193-9)
Supplement: Supplementary file 2 — Table S1. Blastocyst development rates at day 7 in SOF and N2B27. Table S2. Blastocyst development rates at day 8. Table S3. Blastocyst development rates of bovine embryos cultured in low doses of PD0325901. Table S4. Blastocyst development rates of bovine embryos cultured in high doses of PD0325901. Table S5. Blastocyst development rates of bovine embryos cultured in t2iGö+LIF. Table S6. Effect of Wnt and MEK inhibitors on bovine blastocyst development. (DOCX 38 kb) [file 12861_2019_193_MOESM2_ESM.docx]

**Additional File 2 (Canizo *et al*.,)**

**TABLE S1**

**Blastocyst development rates at day 7 cultured in SOF and N2B27**

| Medium | N | Cleavage | Total BLd7 | (%) | Categories | | | |
| --- | --- | --- | --- | --- | --- | --- | --- | --- |
|  |  |  |  |  | **BL** | **(%)** | **BHing and**  **BHed** | **(%)** |
| SOF | 103 | 90 | 62 | 69 ± 6^a^ | 53 | 61 ± 7^a^ | 7 | 8 ± 4^a^ |
| N2B27 | 114 | 100 | 65 | 65 ± 6^a^ | 56 | 58 ± 6^a^ | 8 | 8 ± 3^a^ |

Number of bovine embryos cultured from day 5 to 8-9 in N2B27 or SOF (control) and its odds (number of embryos per number of cleavage) in percentage analysed by a Fisher´s exact test. Same letters denote non-significant differences, p>0.05. BL: blastocysts; BHing: blastocyst hatching; BHed: hatched blastocysts.

**TABLE S2**

**Blastocyst development rates at day 8 cultured in SOF and N2B27**

| Medium | N | Cleavage | Total BLd7 | (%) | Categories | | | |
| --- | --- | --- | --- | --- | --- | --- | --- | --- |
|  |  |  |  |  | **BL** | **(%)** | **BHing and**  **BHed** | **(%)** |
| SOF | 47 | 26 | 19 | 51 ± 14^a^ | 7 | 40 ± 7^a^ | 2 | 15 ± 8^a^ |
| N2B27 | 63 | 36 | 19 | 54 ± 13^a^ | 8 | 30 ± 6^a^ | 2 | 13 ± 6^a^ |

Number of bovine embryos cultured from day 5 to 8-9 in N2B27 or SOF (control) and its odds (number of embryos per number of cleavage) in percentage analysed by a Fisher´s exact test. Same letters denote non- significant differences, *p*>0.05. BL: blastocysts; BHing: blastocyst hatching; BHed: hatched blastocysts.

**TABLE S3**

**Blastocyst development rates of bovine embryos cultured in low doses of PD0325901**

| Treatments | n | N | Cleavage | BL (%) | BE (%) | BHing (%) | BHed (%) | Total* (%) |
| --- | --- | --- | --- | --- | --- | --- | --- | --- |
| DMSO | 3 | 82 | 57 | 4  (6 ± 4)^a^ | 13  (22 ± 8)^a^ | 3  (5 ± 3)^a^ | 7  (12 ± 5)^a^ | 27  (50 ± 10)^a^ |
| PD032  0.4 µM | 3 | 84 | 60 | 8  (13 ± 5)^a^ | 10  (16 ± 6)^a^ | 5  (8 ± 4)^a^ | 12  (19 ± 7)^a^ | 35  (60 ± 9)^a^ |
| PD032  1 µM | 3 | 81 | 59 | 9  (14 ± 6)^a^ | 7  (11 ± 5)^a^ | 5  (8 ± 4)^a^ | 9  (15 ± 6)^a^ | 30  (53 ± 10)^a^ |
| PD032  2 µM | 3 | 84 | 55 | 5  (8 ± 4)^a^ | 4  (7 ± 4)^a^ | 5  (9 ± 4)^a^ | 13  (23 ± 7)^a^ | 27  (50 ± 10)^a^ |

Number of bovine embryos cultured from day 5 to 8-9 in N2B27 with MEK/ERK inhibitor PD032 at low doses (0.4; 1 and 2 µM) or DMSO (control). * day 8 production. Values between brackets represent the estimated mean (percentage) ± S.E. per treatment analysed by a GLMM with Binomial distribution and logit as link function. Different letters denote significant differences, p<0.05. BL: blastocysts; BE: expanded blastocysts; BHing: blastocyst hatching; BHed: hatched blastocysts.

**TABLE S4**

**Blastocyst development rates of bovine embryos cultured in high doses of PD0325901**

| Treatments | n | Cleavage | BL (%) | BE (%) | BHing (%) | BHed (%) | Total* (%) |
| --- | --- | --- | --- | --- | --- | --- | --- |
| DMSO | 4 | 63 | 12  (19 ± 2) ^a^ | 7  (11 ± 4)^b^ | 3  (5 ± 3)^b^ | 2  (3 ± 2)^b^ | 24  (38 ± 8)^b^ |
| PD032  2.5 µM | 3 | 49 | 4  (8 ± 4) ^b^ | 7  (14 ± 5)^b^ | 1  (2 ± 2)^b^ | 1  (2 ± 2)^b^ | 13  (26 ± 8)^b^ |
| PD032  5 µM | 3 | 46 | 5  (11 ± 5) ^b^ | 11  (24 ± 6) ^a^ | 4  (9 ± 4)^b^ | 2  (4 ± 3)^b^ | 22  (46 ± 10)^b^ |
| PD032  10 µM | 3 | 18 | 4  (22 ± 10) ^a^ | 5  (28 ± 11)^a^ | 3  (17 ± 9)^a^ | 2  (11 ± 8)^a^ | 14  (81 ± 10)^a^ |

Number of bovine embryos cultured from day 5 to 8-9 in N2B27 with PD0325901 at high doses or in DMSO (control vehicle).* Day 8 production. Values between brackets represent the estimated mean ± S.E. Different letters denote significant differences, *p*<0.05 in a GLMM analysis with a Binomial distribution and logit as link function. BL: blastocysts; BE: expanded blastocysts; BHing: blastocyst hatching; BHed: hatched blastocysts.

**TABLE S5**

**Blastocyst development rates of bovine embryos cultured in t2iGö+LIF**

| Treatments | n | N | Cleavage | BL (%) | BE (%) | BHing (%) | BHed (%) | Total* (%) |
| --- | --- | --- | --- | --- | --- | --- | --- | --- |
| DMSO | 3 | 111 | 78 | 1  (1 ± 1) | 0  (0 ± 0) | 0  (0 ± 0) | 18  (23 ± 5) | 19  (24 ± 5)^c^ |
| t2iGö | 4 | 244 | 155 | 4  (2 ± 1) | 0  (0 ± 0) | 2  (2 ± 1) | 17  (11 ± 3) | 23  (15 ± 3)^b^ |
| h-LIF | 4 | 131 | 100 | 1  (1 ± 1) | 3  (3 ± 2) | 0  (0 ± 0) | 18  (18 ± 4) | 22  (22 ± 4)^a^ |
| t2iGö h-LIF | 3 | 250 | 185 | 5  (2 ± 1) | 8  (4 ± 1) | 1  (2 ± 1) | 45  (24 ± 3) | 59  (32 ± 3)^a^ |

Number of bovine embryos cultured from day 5 to 8-9 in N2B27 with t2iGö, h-LIF, t2iGö + h-LIF or DMSO (control). * development assessed on day 8. Values between brackets represent the estimated mean ± S.E. per treatment. Different letters denote significant differences, *p*<0.05 in a GLMM analysis with Binomial distribution and logit as link function. BL: blastocysts; BE: expanded blastocysts; BHing: blastocyst hatching; BHed: hatched blastocysts.

**TABLE S6**

**Effect of Wnt and MEK inhibitors on bovine blastocyst development**

| Treatments | n | Cleavage | BL (%) | BE (%) | BHing (%) | BHed (%) | Total* (%) |
| --- | --- | --- | --- | --- | --- | --- | --- |
| DMSO | 5 | 97 | 17  (18 ± 4) | 8  (9 ± 3) | 6  (6 ± 2) | 10  (8 ± 4) | 41  (43 ± 6)^b^ |
| IWP2 | 4 | 83 | 7  (8 ± 3) | 17  (18 ± 5) | 0  (0 ± 0) | 2  (3 ± 2) | 26  (29 ± 6)^c^ |
| PD032  10 µM | 3 | 51 | 8  (16 ± 5) | 6  (13 ± 6) | 6  (12 ± 5) | 12  (15 ± 7) | 32  (64 ± 8)^a^ |
| IWP2 + PD032 | 3 | 36 | 3  (8 ± 5) | 12  (35 ± 9) | 0  (0 ± 0) | 5  (11 ± 6) | 20  (57 ± 9)^a^ |

Number of bovine embryos cultured from day 5 to 8-9 in N2B27 with Wnt inhibitor (IWP2), MEK inhibitor (PD032), IWP2 + PD032 or DMSO (control).* Day 8 production. Values between brackets represent the estimated mean ± S.E. per treatment. Different letters denote significant differences, *p*<0.05 in a GLMM analysis with Binomial distribution and logit as link function. BL: blastocysts; BE: expanded blastocysts; BHing: blastocyst hatching; BHed: hatched blastocysts.
